# Supplementary material for: Plant Serpins: Potential Inhibitors of Serine and Cysteine Proteases with Multiple Functions
Source: Plants (Basel). 2023 Oct 19;12(20):3619. doi: 10.3390/plants12203619 (PMC10609998; doi:10.3390/plants12203619)
Supplement: Supplementary file 1 [file plants-12-03619-s001.zip › Table S6.pdf]

PRISMA 2009 Checklist

Table S6. PRISMA checklist.

Section/topic		Checklist item	Reportedon page
**TITLE**			
Title	1	Plant serpins, potential inhibitors of serine and cysteine-proteases with multiple functions.	Title page, Page 1
**ABSTRACT**			
Structured summary	2	Serpins are a superfamily of protein inhibitors found in all kingdoms of life and are mainly characterized as serine-protease inhibitors. Interest in research involving plant serpins has been instigated primarily by the biotechnological potential related to human diseases demonstrated by many serpins. However, despite ongoing studies with these inhibitors, the biological role of this family in the plant kingdom has not yet been fully clarified. In order to obtain new insights into the potential of plant serpins, this study presents the first systematic review of the topic. We selected and summarized data from studies indexed in different databases, using a search string and previously defined inclusion and exclusion criteria. So far, many narrative reviews have been performed to synthesize the knowledge about the superfamily. However, no systematic review with serpins from the plant kingdom has been performed. The main objective of this study was to scrutinize the published literature in order to increase the knowledge about the proteins in this superfamily. Here, based on the eligible studies, we point out the inhibitory functions of serpins against different serine and non-serine proteases in plants, animals, and related pathogens. The system biology analysis clearly demonstrates that serpins are related to different biological pathways, such as proteolysis control, cell regulation, pollen development, catabolism, and protein dephosphorylation. Moreover, the systematization of the data further contributes to profile the expression of serpins when plants are affected by varied biotic and abiotic stresses, resulting in over-expression or under-expression. We also highlight the serpins, such as AtSerpin1, *OSP-LRS*, *MtSer6*, *AtSRP4*, *AtSRP5* and *MtPiI4*, that influence plant resistance and contribute to increase or reduce cell death. The information systematized here contributes to the design of new studies of plant serpins, especially those aimed at exploring their biotechnological potentials.	Abstract, Page 1
**INTRODUCTION**			
Rationale	3	The information compiled in this systematic review highlights pertinent questions about plant serpins, their expression pattern, functionality and biotechnological potential, and adds knowledge so that future research can focus on questions that still need to be answered.	Introduction, Page 1
Objectives	4	The systematic review was prepared based on a properly established protocol and with the objective of expanding and systematizing the knowledge about plant serpins. The questions that supported the review were formulated based on the PICOS strategies and discussed by peers.	Materials and Methods, in section 4.1 Planning Page 17
**METHODS**			
Protocol and registration	5	The systematic review was performed using the keywords determined according to the protocol and connected by the AND connector. The asterisk (*) was used to assist in the terminal variations of the search words. The resulting search string used in all databases was represented by the words: serpin* AND plant*. Only articles in English and containing experimental data were accepted.	Table S4
Eligibility criteria	6	Only articles in English with experimental results involving plant serpins were selected.	Table S4

Information sources	7	Searches were performed in the Scopus, Web of Science and PubMed databases. The files were imported in the BIBTEX format, compatible with the StArt software. The surveys were conducted from May 2020 to April 2022.	Materials and Methods, in section 4.2: Execution<br>Page 17
Search	8	The searches performed in the three databases (Scopus, Web of Science and PubMed) used the keywords, added to the "advanced" search bar, and using the conjunction "AND". The searches considered only the title, abstract, and keywords in each database. The files were downloaded in BibTex format, except for those from PubMed, which were exported in CSV format and converted to BibTex format using the Zotero (6.0.15) software. Files in BibTex format were exported to the StArt software.	Materials and Methods, in section 4.2: Execution<br>Page 17
Study selection	9	The search for articles in the selection stage was carried out in the selected databases, and considered only the reading of titles, abstracts and keywords.	Materials and Methods, in section 4.2: Execution<br>Page 17
Data collection process	10	Only articles that met the inclusion criteria and answered at least one of the questions were accepted for the review. At this stage, the articles were read in full, and they were analyzed regarding the following questions:<br>1. In which countries has research with plant serpins been conducted?<br>2. Among the serpins identified, which ones were characterized or presented more comprehensive studies, and in which species?<br>3. In which plant parts have the serpins been identified?<br>4. Are plant serpins serine or cysteine protease inhibitors? And against which proteases do serpins have inhibitory potential?<br>5. Do serpins have other functions besides the inhibitory ones?<br>6. Can serpins be related to defense events and plant stresses?<br>7. Have the biological routes and functions of serpins been well established?	Materials and Methods, in section 4.2: Execution<br>Page 17;<br>Table S4
Data items	11	Plant serpins, countries of study on the subject, level of location and expression, functional characterization, inhibitory potential, stresses and biological processes.	Table S4
Risk of bias in individual studies	12	The selection of articles followed the inclusion and exclusion criteria.	Materials and Methods, in section 4.1: Planning<br>Page 17;<br>Table S4
Summary measures	13	We did not employ meta-analyses in the study.	N/A
Synthesis of results	14	We did not employ meta-analyses in the study.	N/A

PRISMA 2009 Checklist

Section/topic		Checklist item	Reportedon page
Risk of bias across studies	15	The review respected the pre-established inclusion and exclusion criteria, and the formulation of the guiding questions was based on the PICOS guidelines.	Materials and Methods, in section 4.1: Planning Page 17; Table S5
Additional analyses	16	No additional analysis method was used.	N/A
**RESULTS**			
Study selection	17	Electronic searches in the selected databases (Scopus, Web of Science and PubMed) were performed with the help of the StArt software, and a total of 533 articles were returned. The Scopus database contributed the most papers, a total of 280 articles, followed by Web of Science with 147 and PubMed with 106 articles. After initial analysis, 429 papers were removed. Of these, 230 were duplicates and 199 were not in accordance with the pre-established criteria according to the protocol. The remaining 104 articles were submitted to full reading and evaluated for eligibility. At the end of the analysis, 90 papers were accepted for being in accordance with all the established criteria (inclusion and exclusion), and for answering the questions proposed in the protocol.	Results, Pages 2, 3
Study characteristics	18	The relevant information for the composition of the review was extracted according to the questions proposed in the protocol.	Table S4
Risk of bias within studies	19	No paper was selected in the presence of the exclusion criteria.	Table S4
Results of individual studies	20	The individual results obtained based on the summarized results are presented in tables and figures.	
Synthesis of results	21	No meta-analysis was used.	N/A
Risk of bias across studies	22	No risk analysis was performed. Possible biases were mitigated only with the use of inclusion and exclusion criteria, and a complete reading of the studies, in which the applied methodology was analyzed, and whether the experimental designs were able to achieve the results obtained.	Table S4
Additional analysis	23	No additional methods of analysis were used.	N/A
**DISCUSSION**			
Summary of evidence	24	- Serpin inhibitors have been studied in several countries. China, Denmark, USA and Israel are the most prominent countries in research of the area.<br>- Serpins are ubiquitously located proteins in different species.<br>- Serpins are inhibitors of different serine proteases in animals, and also of cysteine proteases in plants.<br>- They are proteins related to the control of proteolysis, phase transition and regulation of the cell cycle, pollen development, catabolism and dephosphorylation of proteins.<br>- They are defense proteins and are involved in different biotic and abiotic stresses.<br>- The overexpression or underexpression of serpins in transgenic plants reduces or increases cell death caused by stress, respectively.	Results, Pages 2-14

Limitations	25	Although there are a considerable number of studies involving plant serpins, much about this superfamily of plant inhibitors still remains unknown, and there is also a lack of knowledge characterizing the biological routes and the action of these inhibitors in the plant kingdom and in pathosystems.	Conclusion, Page 18
Conclusions	26	The systematization of the data presented in this review has outlined the current state of research involving serpins. The serpins are ubiquitously present in many plant species. These proteins are mainly characterized as inhibitors of serine proteases, except for two serpins, AtSerpin1 and CrSERPIN, which are capable of inhibiting cysteine proteases. In addition, they are a superfamily of proteins that exhibit multiple functions related to defense, cell death, RNA binding, and chaperone activity, giving them outstanding biotechnological potential, especially functionally characterized ones.<br>This systematic review identifies the considerable information accumulated about plant serpins in recent years and offers knowledge so that future research can achieve results with greater applicability and scientific guidance. Many of the serpins listed here have not yet been characterized. This is a relevant point since many of these proteins can be targets of new studies.<br>Despite all the scientific efforts described in this review, there is consensus that much about this superfamily of plant inhibitors still remains unknown. Questions about which endogenous or pathogenic serine proteases are affected, or how these proteins act in the regulation of plant survival and defense mechanisms, have not yet been fully clarified. So, further research is needed to clarify doubts and resolve unanswered questions about the functional profile of the serpin superfamily in the plant kingdom.	Conclusion, Page 18
**FUNDING**			
Funding	27	This work was supported by a PhD scholarship granted to M.M.F. by CAPES (Coordenação de Aperfeiçoamento de Pessoal de Nível Superior).	Funding, Page 18

From: Moher D, Liberati A, Tetzlaff J, Altman DG, The PRISMA Group (2009). Preferred Reporting Items for Systematic Reviews and Meta-Analyses: The PRISMA Statement. PLoS Med 6(7): e1000097. doi:10.1371/journal.pmed1000097

For more information, visit: www.prisma-statement.org.
