# Supplementary material for: Plant Serpins: Potential Inhibitors of Serine and Cysteine Proteases with Multiple Functions
Source: Plants (Basel). 2023 Oct 19;12(20):3619. doi: 10.3390/plants12203619 (PMC10609998; doi:10.3390/plants12203619)
Supplement: Supplementary file 1 [file plants-12-03619-s001.zip › Table S1.pdf]

Table S1. Protocol established for conducting the systematic review, according to the PRISMA guidelines [42].

Protocol	
**Title**	
Plant serpins, potential inhibitors of serine and cysteine proteases with multiple functions	
**Objectives**	
- To broaden the knowledge about the serpin superfamily in plants, their identities and localization profiles;<br><br>- To know which proteases are potentially inhibited by these inhibitors and if the serpins have other functions besides inhibitory potential.	
**Keywords: Database/ Search Strings**	
PubMed: (serpin*) AND plant*[Title/Abstract]<br>Scopus: TITLE-ABS-KEY (serpin* AND plant*)<br>Web of Science: TS=(serpin* AND plant*)	
**Questions**	
1. In which countries has research with plant serpins been conducted?<br>2. Among the serpins identified, which ones were characterized or presented more comprehensive studies, and in which species?<br>3. In which plant parts have the serpins been identified?<br>4. Are plant serpins serine or cysteine protease inhibitors? And against which proteases do serpins have inhibitory potential?<br>5. Do serpins have other functions besides the inhibitory ones?<br>6. Can serpins be related to defense events and plant stresses?<br>7. Have the biological routes and functions of serpins been well established?	
**Study selection criteria**	
**Inclusion**	**Exclusion**
- Research articles with experimental results only with plant serpins<br><br>- Articles published in English	- Works such as dissertations and theses, books or book chapters, other reviews, protocols or reports, conference annals and short communications.<br><br>- Non-English language article.<br><br>- Inconclusive results or lack of validation demonstrated by figures, graphs, tables etc.<br><br>- Articles that did not answer any of the questions in this review.<br><br>- Non-serpin inhibitors in plants.
