# Supplementary material for: Plant Serpins: Potential Inhibitors of Serine and Cysteine Proteases with Multiple Functions
Source: Plants (Basel). 2023 Oct 19;12(20):3619. doi: 10.3390/plants12203619 (PMC10609998; doi:10.3390/plants12203619)
Supplement: Supplementary file 1 [file plants-12-03619-s001.zip › Table S2.pdf]

Table S2. Articles selected for the Systematic Review.

Title	Authors	DOI
A 39 kd barley seed protein of the serpin superfamily inhibits α-chymotrypsin	[48]	https://doi.org/10.1007/BF02904471
A plant serpin gene. Structure, organization and expression of the gene encoding barley protein Z4	[73]	https://doi.org/10.1111/j.1432-1033.1990.tb15644.x
Major proteins of beer and their precursors in barley: Electrophoretic and immunological studies	[74]	https://doi.org/10.1021/jf00058a013
Inhibition of coagulation factors by recombinant barley serpin BSZx	[15]	https://doi.org/10.1016/0014-5793(96)00940-4
Heterologous expression of three plant serpins with distinct inhibitory specificities	[14]	https://doi.org/10.1074/jbc.271.41.25083
Inhibitory serpins from wheat grain with reactive centers resembling glutamine-rich repeats of prolamin storage proteins. Cloning and characterization of five major molecular forms	[16]	https://doi.org/10.1074/jbc.M004633200
Degradation of endosperm mRNAs during dry after ripening of cereal grains	[75]	https://doi.org/10.1017/S096025850000026X
Characterization of *Cucurbita maxima* phloem serphin-1 (CmPS-1). A developmentally regulated elastase inhibitor	[51]	https://doi.org/10.1074/jbc.M006060200
Inhibitory serpins from rye grain with glutamine as P1 and P2 residues in the reactive center	[49]	https://doi.org/10.1016/S0014-5793(00)02425-X
Serpins of oat (*Avena sativa*) grain with distinct reactive centers and inhibitory specificity	[50]	https://doi.org/10.1034/j.1399-3054.2002.1160204.x
Differential gene expression for suicide-substrate serine proteinase inhibitors (serpins) in vegetative and grain tissues of barley	[61]	https://doi.org/10.1093/jxb/erg248
Altered gene expression in three plant species in response to treatment with Nep1, a fungal protein that causes necrosis	[76]	https://doi.org/10.1104/pp.102.019836
The barley starch granule proteome - Internalized granule polypeptides of the mature endosperm	[77]	https://doi.org/10.1016/j.plantsci.2003.10.028
Putative protease inhibitor gene discovery and transcript profiling during fruit development and leaf damage in grapefruit (*Citrus paradisi* Macf.)	[78]	https://doi.org/10.1016/j.gene.2003.10.010
Proteome analysis of barley seeds: Identification of major proteins from two-dimensional gels (p/ 4-7)	[79]	https://doi.org/10.1002/pmic.200300753
The identification of foam-forming soluble proteins from wheat (*Triticum aestivum*) dough	[80]	https://doi.org/10.1002/pmic.200401035
Serpins in fruit and vegetative tissues of apple (*Malus domestica*): Expression of four serpins with distinct reactive centres and characterisation of a major inhibitory seed form, MdZ1b	[52]	https://doi.org/10.1071/FP04220
Probing heat-stable water-soluble proteins from barley to malt and beer	[81]	https://doi.org/10.1002/pmic.200401153
Cucurbit phloem serpins are graft-transmissible and appear to be resistant to turnover in the sieve element-companion cell complex	[82]	https://doi.org/10.1093/jxb/eri308
Serpin1 of *Arabidopsis thaliana* is a Suicide Inhibitor for Metacaspase 9	[43]	https://doi.org/10.1016/j.jmb.2006.09.010
Is quantity of protein in barley forms determined by proteins localized in the subaleurone layer?	[83]	https://doi.org/10.1007/BF02706623
Proteomic analysis of wheat flour allergens	[54]	https://doi.org/10.1021/jf070843a
A comprehensive analysis of the 14-3-3 interactome in barley leaves using a complementary proteomics and two-hybrid approach	[84]	https://doi.org/10.1104/pp.106.090159

Proteomic analysis of wheat proteins recognized by IgE antibodies of allergic patients	[55]	https://doi.org/10.1002/pmic.200700347
Effect of environmental stress during grain filling on the soluble proteome of wheat (*Triticum aestivum*) dough liquor	[85]	https://doi.org/10.1021/jf800209b
Serpin genes *AtSRP2* and *AtSRP3* are required for normal growth sensitivity to a DNA alkylating agent in *Arabidopsis*	[86]	https://doi.org/10.1186/1471-2229-9-52
Integration of the barley genetic and seed proteome maps for chromosome 1H, 2H, 3H, 5H and 7H	[87]	https://doi.org/10.1007/s10142-008-0101-z
Lipid transfer proteins and protease inhibitors as key factors in the priming of barley responses to Fusarium head blight disease by a biocontrol strain of *Pseudomonas fluorescens*	[88]	https://doi.org/10.1007/s10142-010-0177-0
Les Maîtres de l'Orge: The Proteome Content of Your Beer Mug	[89]	https://doi.org/10.1021/pr100551n
Arabidopsis AtSerpin1, crystal structure and in vivo interaction with its target protease RESPONSIVE to DESICCATION-21 (RD21)	[44]	https://doi.org/10.1074/jbc.M109.095075
Proteome analysis of Fusarium head blight in grains of naked barley (*Hordeum vulgare* subsp. nudum)	[90]	https://doi.org/10.1002/pmic.201000322
Potential use of a serpin from *Arabidopsis* for pest control	[29]	https://doi.org/10.1371/journal.pone.0020278
Multiple wheat flour allergens and cross-reactive carbohydrate determinants bind IgE in baker's asthma	[56]	https://doi.org/10.1111/j.1398-9995.2011.02636.x
Mapping of quantitative trait loci associated with protein expression variation in barley grains	[91]	https://doi.org/10.1007/s11032-010-9432-2
Differential effects of a post-anthesis fertilizer regimen on the wheat flour proteome determined by quantitative 2-DE	[92]	https://doi.org/10.1186/1477-5956-9-46
Development of DNA markers associated with beer foam stability forbarley breeding	[38]	https://doi.org/10.1007/s00122-010-1436-0
Bioactive compounds obtained by immobilisation of serine protease inhibitors	[93]	https://doi.org/10.1504/IJNBM.2011.045884
Analyses of albumins, globulins and amphiphilic proteins by proteomic approach give new insights on waxy wheat starch metabolism	[94]	https://doi.org/10.1016/j.jcs.2010.11.001
Serpins in rice: Protein sequence analysis, phylogeny and gene expression during development	[95]	https://doi.org/10.1186/1471-2164-13-449
Proteome analysis of gut and salivary gland proteins of fifth-instar nymph and adults of the sunn pest, *Eurygaster integriceps*	[59]	https://doi.org/10.1002/arch.21047
Phosphatase activity in barley proteins tightly bound to DNA and its development-dependent changes	[96]	https://doi.org/10.1134/S0006297912060168
Molecular and immunological characterization of wheat Serpin (Tri a 33)	[97]	https://doi.org/10.1002/mnfr.201200244
Distribution of alleles of grain quality genes in Indian bread wheat varieties	[98]	No DOI
Set-point control of RD21 protease activity by AtSerpin1 controls cell death in *Arabidopsis*	[17]	https://doi.org/10.1111/tpj.12141
Proteomic analysis of differences in barley (*Hordeum vulgare*) malts with distinct filterability by DIGE	[30]	http://dx.doi.org/10.1016/j.jprot.2013.05.038
Differences in grain ultrastructure, phytochemical and proteomic profiles between the two contrasting grain Cd-accumulation barley genotypes	[99]	https://doi.org/10.1371/journal.pone.0079158
iTRAQ-based quantitative proteome and phosphoprotein characterization reveals the central metabolism changes involved in wheat grain development	[100]	https://doi.org/10.1186/1471-2164-15-1029
Characterizaiton of barley serpin Z7 that plays multiple roles in malt and beer	[31]	https://doi.org/10.1021/jf405699z

A proteinaceous fraction of wheat bran may interfere in the attachment of enterotoxigenic E. coli K88 (F4+) to porcine epithelial cells	[101]	https://doi.org/10.1371/journal.pone.0104258.
Specific nongluten proteins of wheat are novel target antigens in celiac disease humoral response	[57]	https://doi.org/10.1021/pr500809b
Rising CO2 concentration altered wheat grain proteome and flour rheological characteristics	[102]	https://doi.org/10.1016/j.foodchem.2014.07.044
Proteomics, peptidomics, and immunogenic potential of wheat beer (weissbier)	[103]	https://doi.org/10.1021/acs.jafc.5b00631
Proteomic analysis reveals key proteins and phosphoproteins upon seed germination of wheat (*Triticum aestivum* L.)	[104]	https://doi.org/10.3389/fpls.2015.01017
Mechanisms of resistance/tolerance of *Pyrus communis* to *Stemphylium vesicarium*. A transcriptome analysis	[105]	https://doi.org/10.1007/s10457-015-9831-9
Ectopic expression of a proteinase Inhibitor I4 (MtPiI4) Gene from *Medicago truncatula* confers plant resistance to *Pseudomonas syringae* pv. Tomato DC3000	[65]	https://doi.org/10.1007/s11105-015-0865-y
Down-regulation of rice serpin gene *OsSRP-LRS* exaggerates stress-induced cell death	[19]	https://doi.org/10.1007/s12374-015-0283-6
Transcript profiling of serine- and cysteine protease inhibitors in *Triticum aestivum* varieties with different drought tolerance	[106]	https://doi.org/10.1556/0806.43.2015.032
Three sorghum serpin recombinant proteins inhibit midgut trypsin activity and growth of corn earworm	[32]	https://doi.org/10.1016/j.aggene.2016.09.005
The SBT6.1 subtilase processes the GOLVEN1 peptide controlling cell elongation	[107]	https://doi.org/10.1093/jxb/erw241
Singlet oxygen-induced membrane disruption and serpin-protease balance in vacuolar-driven cell death	[45]	https://doi.org/10.1104/pp.15.02026
Identification of drought stress related proteins from 1Sl(1B) chromosome substitution line of wheat variety Chinese Spring	[108]	https://doi.org/10.1186/s40529-016-0134-x
Effects of post-anthesis fertilizer on the protein composition of the gluten polymer in a US bread wheat	[109]	https://doi.org/10.1016/j.jcs.2015.12.002
Distinct metabolic changes between wheat embryo and endosperm duringgrain development revealed by 2D-DIGE-based integrative proteomeanalysis	[110]	https://doi.org/10.1002/pmic.201500371
Comparative proteomic analysis of two barley cultivars (*Hordeum vulgare* L.) with Contrasting Grain Protein Content	[111]	https://doi.org/10.3389/fpls.2016.00542
The study of plant protein accumulation in gut of insect using proteomics technique: Wheat–sunn pest interaction	[112]	https://doi.org/10.1016/j.jssas.2015.06.005
The effect of *Fusarium culmorum* infection and deoxynivalenol (DON) application on proteome response in barley cultivars Chevron and Pedant	[113]	https://doi.org/10.1016/j.jprot.2017.07.005
Serpin1 and WSCP differentially regulate the activity of the cysteine protease RD21 during plant development in *Arabidopsis thaliana*	[46]	https://doi.org/10.1073/pnas.1621496114
Comparative proteomic analysis of two transgenic low-gliadin wheat lines and non-transgenic wheat control	[114]	https://doi.org/10.1016/j.jprot.2017.06.010
Arabidopsis thaliana serpins AtSRP4 and AtSRP5 negatively regulate stress-induced cell death and effector-triggered immunity induced by bacterial effector *AvrRpt2*	[37]	https://doi.org/10.1111/ppl.12516
The tomato hybrid proline-rich protein regulates the abscission zone competence to respond to ethylene signals	[115]	https://doi.org/10.1038/s41438-018-0033-2

Singlet oxygen plays an essential role in the root's response to osmotic stress	[116]	https://doi.org/10.1104/pp.18.00634
Proteomic analysis of the impacts of powdery mildew on wheat grain	[117]	https://doi.org/10.1016/j.foodchem.2018.04.024
Noncanonical interactions between serpin and β-amylase in barley grain improve β-amylase activity *in vitro*	[22]	https://doi.org/10.1002/pld3.54
Host cell proteome of *Physcomitrella patens* harbors proteases and protease inhibitors under bioproduction conditions	[118]	https://doi.org/10.1021/acs.jproteome.8b00423
Drought-induced senescence of *Medicago truncatula* nodules involves serpin and ferritin to control proteolytic activity and iron levels	[20]	https://doi.org/10.1111/nph.15298
AtSERPIN1 is an inhibitor of the metacaspase AtMC1-mediated cell death and autocatalytic processing in planta	[18]	https://doi.org/10.1111/nph.14446
Analysis of the natural dehydration mechanism during middle and latestages of wheat seeds development by some physiological traits and iTRAQ-based proteomic	[119]	https://doi.org/10.1016/j.jcs.2017.12.015
Serpins: Genome-wide characterisation and expression analysis of the serine protease inhibitor family in *Triticum aestivum*	[120]	https://doi.org/10.1534/g3.119.400444
Hordein accumulation in developing barley grains	[121]	https://doi.org/10.3389/fpls.2019.00649
Construction of a comprehensive beer proteome map using sequential filter-aided sample preparation coupled with liquid chromatography tandem mass spectrometry	[39]	https://doi.org/10.1002/jssc.201900074
Comparative analyses of albumin/globulin grain proteome fraction in differentially salt-tolerant Tunisian barley landraces reveals genotype-specific and defined abundant proteins	[122]	https://doi.org/10.1111/plb.12965
Detection and in vitro studies of *Cucurbita maxima* phloem serpin-1 RNA-binding properties	[23]	https://doi.org/10.1016/j.biochi.2020.01.006
Genome wide identification and comparative analysis of the serpin gene family in *Brachypodium* and Barley	[123]	https://doi.org/10.3390/plants9111439
Endopeptidases, exopeptidases, and glutamate decarboxylase in soybean water extract and their in vitro activity	[124]	https://doi.org/10.1016/j.foodchem.2021.130026
RNA binding by plant serpins *in vitro*	[58]	https://doi.org/10.1134/S0006297921100059
Plant type I metacaspases are proteolytically active proteases despite their hydrophobic nature	[47]	https://doi.org/10.1002/1873-3468.14165
EMS derived wheat mutant BIG8-1 (*Triticum aestivum* L.) — A new drought tolerant mutant wheat line	[125]	https://doi.org/10.3390/ijms22105314
ApSerpin-ZX from *Agapanthus praecox*, is a potential cryoprotective agent to plant cryopreservation	[53]	https://doi.org/10.1016/j.cryobiol.2020.11.018
Proteomics unravels new candidate genes of *Dasypyrum villosum* for improving wheat quality	[28]	https://doi.org/10.1016/j.jprot.2021.104292
Shotgun proteomics coupled to transient-inducible gene silencing revealrice susceptibility genes as new sources for blast disease resistance	[126]	https://doi.org/10.1016/j.jprot.2021.104223
