# Supplementary material for: Plant Serpins: Potential Inhibitors of Serine and Cysteine Proteases with Multiple Functions
Source: Plants (Basel). 2023 Oct 19;12(20):3619. doi: 10.3390/plants12203619 (PMC10609998; doi:10.3390/plants12203619)
Supplement: Supplementary file 1 [file plants-12-03619-s001.zip › Table S3.pdf]

Table S3. Distribution of serpins in different parts of the plant.

Serpins**	Localization	Author
	Hordeum vulgare	
BSZ7 (Serpin-Z7) (Q43492.2)	Seeds/Grains	[48]<br>[79]*<br>[87]*<br>[30]*<br>[99]*<br>[31]*<br>[111]*<br>[113]*<br>[122]*<br>[121]*
	Seeds/Grains, embryos, embryo-derived callus, coleoptiles, shoots, roots	[61]
	Seeds/Grains, beer	[38]
	Beer	[89]*<br>[103]*
BSZ4 (Serpin-Z4) (P06293.2)	Seeds/Grains	[73]<br>[77]*<br>[79]*<br>[90]*<br>[30]*<br>[111]*<br>[121]*
	Seeds/Grains, embryos, embryo-derived callus, roots, coleoptiles, leaves, shoots and senescing leaves,	[61]
	Seeds/Grains, beer	[38]
	Seeds/Grains, cytosol of transformed tobacco leaves	[22]
	Beer	[89]*<br>[39]*<br>[103]*
	Barley heads	[88]
Z-type serpin (CAA66232.1)	Seeds/Grains	[83]*<br>[91]*<br>[90]*<br>[99]*<br>[113]*
	Seeds/Grains, beer	[74]<br>[81]*
BSZx (Serpin-Zx) (Q40066.1)	Seeds/Grains	[79]*<br>[87]*<br>[121]*<br>[122]*
	Seeds/Grains, embryos, embryo-derived callus, roots, coleoptiles, shoots, leaf	[61]
	Beer	[89]*
SPZ5: Putative serpin-Z5 from *Oryza sativa* (Q53MD3)	Barley shoots (first leaves)	[96]*
Serpin (TC No.151425)	Leaf	[84]*
vSRP3-2 (HORVU3Hr1G074320)<br>HvSRP4-1 (HORVU4Hr1G013480)<br>HvSRP5-1 (HORVU5Hr1G101710)	Grain (caryopsis)	[123]

HvSRP5-2 (HORVU5Hr1G111860)<br>HvSRP5-3 (HORVU5Hr1G111920)<br>HvSRP6-1 (HORVU6Hr1G008650)<br>HvSRP6-2 (HORVU6Hr1G008840)		
HvSRP1-1 (HORVU1Hr1G071460)	Root seedling, shoot seedling, internodal area	
HvSRP7-1 (HORVU7Hr1G008620)	Root seedling and internodes, embryo	
HvSRP4-3 (HORVU4Hr1G013550)<br>HvSRP4-7 (HORVU4Hr1G079160)<br>HvSRP6-3 (HORVU6Hr1G008860)	Germinating embryo	
HvSRP4-6 (HORVU4Hr1G064290)	Caryopsis, embryo	
Triticum aestivum L.		
WSZ1a (Serpin-Z1A) (Q41593.1)	Seeds/Grains	[16]<br>[80]*<br>[94]*<br>[114]*<br>[117]*<br>[85]*<br>[59]*<br>[104]*<br>[119]*<br>[28]*
	Seeds/Grains, ovary, rachis, spike	[120]
	wheat flour	[57]*
	Beer	[103]*
WSZ1b (Serpin-Z1B) (P93693)	Seeds/Grains	[16]<br>[80]*<br>[92]*<br>[114]*<br>[119]*
	Seeds/Grains, ovary, rachis, spike	[120]
	Wheat flour	[54]*<br>[55]*
	Beer	[103]*
WSZ1c (Serpin-Z1C) (Q9ST58)	Seeds/Grains	[16]<br>[92]*<br>[102]*<br>[104]*<br>[119]*<br>[28]*
	Seeds/Grains, rachis, spike	[120]
	wheat flour	[54]*<br>[57]*
	Beer	[103]*
WSZ2a (Serpin-Z2A) (Q9ST57)	Seeds/Grains	[16]<br>[80]*<br>[100]*<br>[114]*<br>[110]*<br>[28]*

	Seeds/Grains, ovary, rachis, spike	[120]
	Beer	[103]*
	Leaves	[125]*
WSZ2b (Serpin-Z2B)<br>(P93692)	Seeds/Grains	[16]<br>[80]*<br>[85]*<br>[94]*<br>[92]*<br>[104]*<br>[108]*<br>[110]*<br>[114]*<br>[28]*
	Seeds/Grains, ovary, rachis, spike	[120]
	Wheat bran	[101]*
	Wheat flour	[54]*<br>[57]*
	Beer	[103]*
	Leaves	[125]*
WSZ2c	Seeds/Grains, ovary, rachis, spike	[120]
TraesCS3B01G335800<br>TraesCS4A01G422200<br>TraesCS4B01G079100<br>TraesCS6D01G048700<br>TraesCS3D01G301100<br>TraesCS6D01G114700<br>TraesCS2B01G530600	Seeds/Grains	[120]
TraesCS4B01G079200<br>TraesCS4A01G235700	Seeds/Grains, spike, rachis, ovary	
TraesCS4A01G436000<br>TraesCS4A01G205200<br>TraesCS4D01G231200	Seeds/Grains, spike	
TraesCS6B01G068900<br>TraesCS4D01G106100	Seeds/Grains, spike, ovary	
Serpin 1 (Serpin Bu-1)<br>(C0LF30)	Seeds/Grains	[92]*<br>[114]*<br>[117]*<br>[110]*<br>[104]*
	Leaves, roots	[106]
	Plants (it was not reported which part of the plant was used for feeding the insects)	[112]*
Serpin 2<br>(C0LF31)	Seeds/Grains	[100]*<br>[110]*
	Leaves, roots	[106]
Serpin 3<br>(C0LF32)	Seeds/Grains	[100]*
	wheat flour	[57]*
Serpin-N3.2<br>(H9AXB3)	Seeds/Grains	[100]*<br>[110]*
Serpin Bu-4	Seeds/Grains	[92]*
Srp5B		[98]*
Serpin (unreported identity)	Seeds/Grains	[109]*
Serpin<br>(A0A3B6TLW2)	Seeds/Grains	[119]*<br>[28]*
SERPIN domain-containing protein<br>(A0A3B6MWJ8)	Seeds/Grains	[119]*<br>[28]*

A0A3B6DK48<br>A0A3B6HVL4<br>A0A3B6IPZ0<br>A0A3B6JDS9<br>A0A3B6KQC8<br>A0A3B6KQL2<br>A0A3B6KR02<br>A0A3B6KSZ6<br>A0A3B6SDG4<br>A0A3B6SF67	Seeds/Grains	[28]*
	Secale cereale	
RSZ (a, b, c1, c2, d, e, f)	Seeds/Grains	[49]
	Avena sativa L.	
OSZ (a, b, c, d)	Seeds/Grains	[50]
	Oryza sativa	
OsSRP-LRS<br>(Os03g41419)	Seeds/Grains, callus, seedling, roots, stems, leaves	[95]
	Leaf sheath, leaves	[19]
OsSRP-PLP<br>(Os11g11500)<br>*OsSRP-LGC*<br>(Os01g56010)<br>*OsSRP-FLC*<br>(Os11g13540)<br>*OsSRP-FRS*<br>(Os03g41438)<br>*OsSRP-QKG*<br>(Os01g16200)	Seeds/Grains, callus, seedling, roots, stems, leaves	[95]
OsSRP-FAZ<br>(Os11g12460)	Seeds/Grains, callus	
OsSRP-PTY<br>(Os04g45110)	Seeds/Grains, seedling, roots, stems	
OsSRP-ZXA<br>(Q75H81)	Leaves	[126]*
	Avena fatua L.	
Protein Z (Z1)	Seeds/Grains	[75]
	Brachypodium distachyon	
BdSRP2-1<br>(Bradi2g50900)<br>BdSRP4-10<br>(Bradi4g23062)<br>BdSRP4-11<br>(Bradi4g23070)<br>BdSRP4-16<br>(Bradi4g24570)	Seeds, anther	[123]
BdSRP4-1<br>(Bradi4g14070)<br>BdSRP4-5<br>(Bradi4g15390)	Anther	
BdSRP1-3<br>(Bradi1g14740)<br>BdSRP4-7<br>(Bradi4g22020)	Embryo	
BdSRP5-5<br>(Bradi5g16780)	Endosperm, pistil, seed	
BdSRP1-2<br>(Bradi1g14730)	Seed, pistil and embryo	
AtSerpin1<br>(Q9S7T8)	Root tip, subcellular localization in apoplast	[43]
	Siliques, flowers, stem, leaf, roots, seedlings	[86]
	Flowers, siliques, stem, leaf, root	[44]
	Seedling, leaf, subcellular localization in cytoplasm	[17]

	Leaf	[45]
	Seedling, hypocotyl, root, cotyledons, first leaves, hypocotyl (in *Arabidopsis*) and subcellular localization in leaf epidermis of *N. benthamiana*	[107]
	Seedlings, cotyledons of greening seedlings	[46]
	Root	[116]
	Leaves (rosette leaves), and subcellular localization in cytoplasm and nucleo of epidermal *N. benthamiana* cells by Agrobacterium-mediated transient expression	[18]
AtSRP2 (*At2g14540*)	Siliques, Flowers, subcellular localization in nucleus	[86]
AtSRP3 (A*t1g64030*)	Siliques, flowers, subcellular localization in cytosol	
AtSRP4 (*At2g26390*)	Siliques, flowers, stem, leaves, roots, seedlings	[86]
	Leaves	[37]
AtSRP5 (*At2g25240*)	Leaves	
	Cucurbita máxima	
CmPS-1 (AAG02411.1)	Stem (phloem exudate)	[51]
	Phloem (phloem exudate after germination): root–shoot transition region	[82]
	Phloem exudates, subcellular localization in *N. benthamiana* leaf epidermal cells	[23]
	Cucumis sativus	
CsPS-1	Phloem (phloem exudate after germination): root–shoot transition region	[82]
	Malus domestica Borkh.	
MdZ1a	Young fruit, buds, expanding leaves, vascular tissues	[52]
MdZ1b	Vegetative buds, cotyledons, seeds	
MdZ1c	Expanding leaves	
MdZ2a	Fruit, buds, seeds, leaves, vascular tissues	
	Medicago truncatula	
MtPiI4	Seed, root, leaf, stem and flower tissues	[65]
MtSer (*5*, *6*, *8*, *9*, *10*)	Nodules (root)	[20]
	Centaurea maculosa (Spotted knapweed)	
CmSER-1	Leave	[76]
	Citrus paradisi Macf. Cv. Marsh	
serpL-1	Grapefruit flavedo, Young immature and mature leaves	[78]
	Citrullus vulgaris	
Serpin (no identification code)	Seeds	[93]
	Pyrus communis	
Serpin-like protein (JK086945)	Leaves	[105]
JK086934		
	Solanum lycopersicum	
Solyc04g079480.2	Abscission zone tissues after flower removal (R)	[115]
	Glycine max	
Serpin-ZX: XP_003528230.1	Seeds	[124]
Serpin-ZX: XP_003523896.1		
	Agapanthus praecox	
ApSerpin-ZX	Embryogenic callus	[53]
	Physcomitrella patens	
Pp1s22_263V6.1	Moss	[118]*
Pp1s414_23V6.1		
Pp1s61_265V6.1		
Pp1s86_63V6.1		

*Refers to articles in which proteins were identified by proteomic analysis.

** Only serpins located by different experimental analyses or extracted from different tissues were counted. Recombinant serpins were not considered.
