# Supplementary material for: Plant Serpins: Potential Inhibitors of Serine and Cysteine Proteases with Multiple Functions
Source: Plants (Basel). 2023 Oct 19;12(20):3619. doi: 10.3390/plants12203619 (PMC10609998; doi:10.3390/plants12203619)
Supplement: Supplementary file 1 [file plants-12-03619-s001.zip › Table S4.pdf]

Table S4. Description of studies and the serpins involved in different biotic and abiotic stresses.

Serpins	Description	Author*
**Biotic**		
CmPS-1	Increased CmPS-1 in phloem sap was correlated with reduced survival of the aphid *Myzus persicae* in *C. maxima.*	[51]
BSZ4 (*Serpin-Z4*)	Serpin-Z4 transcripts were upregulated in barley head tissue in response to treatment with the biocontrol bacterium *Pseudomonas fluorescens* and/or the pathogenic fungus *Fusarium culmorum*.	[88]
BSZ4<br>Z-Type-Serpin	Proteomic analyses identified three serpins that were upregulated in barley grains artificially and naturally infected with *Fusarium* spp.	[90]
AtSerpin1	Bioassays and artificial diet with transgenic plants overexpressing AtSerpin1 reduced the growth of *Spodoptera littoralis* larvae and caused mortality of *Acyrthosiphon pisum* nymphs.	[29]
	Arabidopsis thaliana leaves overexpressing AtSerpin1 compromised the development of the necrotrophic fungi *Botrytis cinerea* and *Sclerotina sclerotiorum*, and it was accelerated when serpin was absent.	[17]
	Overexpression of ASerpin1 resulted in decrease of cell death caused by the pathogen *Pseudomonas syringae* pv tomato expressing the type III effector *avrRpm1* (*Pto* DC3000 (*avrRpm1*)), compared to mutant and wild-type lines.	[18]
JK086945<br>JK086934	Serpin genes showed higher expression levels in the most resistant/tolerant cultivar of *Pyrus communis* after infection with the fungal pathogen *Stemphylium vesicarium* compared to the susceptible cultivar.	[105]
MtPiI4	Expression of *MtPiI4* was induced by the bacterial pathogen *Pseudomonas syringae* pv. tomato DC3000 strain (*Pst* DC3000) and treatment by methyl jasmonate (MeJA) in *Medicago truncatula*. In addition, transgenic *Arabidopsis thaliana* plants overexpressing *MtPiI4* were more resistant to *Pst* DC3000 and reduced bacterial populations.	[65]
Sbser1<br>Sbser2<br>Sbser3	Reductions between 29-53% of the average weight of *Helicoverpa zea* larvae was observed when the serpins Sbser1, Sbser2 and Sbser3 were added individually to the larval diet on sorghum leaves.	[32]
Z-type serpin<br>BSZ7(Serpin Z7)	Z-type serpin and Serpin-Z7 were upregulated under the effect of an artificial infection by *Fusarium culmorum* and application of the mycotoxin Deoxynivalenol (DON) in cobs of two barley varieties.	[113]
WSZ1a (Serpin-Z1A)<br>Serpin 1	Proteomic analysis revealed that spots from Serpin Z1A and serpin 1 were accumulated most in grains from wheat infected by powdery mildew.	[117]
TraesCS4A01G235700	Negative regulation against the pathogens *Blumeria graminis* and *Puccini striiformis*, and positive against *Zymoseptoria tritici.*	[120]
TraesCS2B01G530600	Negative and positive regulation against fungi *Blumeria graminis* and *Fusarium graminearum*, respectively.	
TraesCS4D01G231200	Negative regulation against *Puccini striiformis* and *Blumeria graminis*.	
TraesCS2B01G033100LC<br>TraesCS2B01G033300LC<br>TraesCS3B01G335800<br>TraesCS3D01G301100<br>TraesCS4A01G422200<br>TraesCS4A01G436000<br>TraesCS4D01G106100<br>TraesCS6B01G068900	Serpin genes were positive regulated in response to the fungal pathogen *Fusarium graminearum.*	
TraesCS4B01G079100	Negative regulation against *Fusarium pseudograminearum.*	
TraesCS4B01G079200	Negative regulation against *Puccini striiformis*	

TraesCS4D01G090600<br>TraesCS6B01G152500<br>TraesCS6D01G114700	Serpin genes were positive regulated in response to PAMP elicitors flg22 and chitin.	
TraesCS4A01G205200<br>TraesCS5B01G492700LC	Serpin genes were positive and negative regulated in response to the Flg22.	
TraesCS5B01G402400<br>TraesCS6D01G048700	Serpin genes were positive regulated in response to chitin.	
OsSRP-ZXA (Q75H81)	Among other proteins, OsSRP-ZXA was upregulated in a comparative proteomic study between two near-isogenic rice lines with a resistant phenotype and susceptible to infection by the fungus *Magnaporthe oryzae* 12 hours after inoculation with the pathogen.	[126]
	Abiotic	
WSZ1a (Serpin-Z1A)<br>WSZ2b (Serpin-Z2B)	Two-dimensional electrophoresis analyses of four wheat varieties grown in polytunnels under hot/dry or cold/humid regimes during the grain filling period identified Serpin-Z1A in three varieties, while Serpin-Z2B was absent or weakly detected regardless of variety.	[85]
AtSRP2<br>*AtSRP3*	Mouse ear cress seedlings exposed to methyl methanesulfonate (MMS), a model alkylating reagent that causes DNA damage, exhibited 5- and 100-fold increased expression of the *AtSRP2* and *AtSRP3* genes, respectively.	[86]
Serpin-1<br>*Serpin-2*	The transcriptional profile of protease inhibitors was evaluated in different winter wheat varieties with drought tolerance. Under drought, expression of the serpins in leaves of the most drought-tolerant variety tended to preserve levels close to controls, whereas in roots, *Serpin-1* exhibited variable expression in different plants, but with higher levels in the most drought-tolerant variety.	[106]
WSZ2b<br>(Serpin-Z2B)	Serpin-Z2B protein was positively regulated under water stress in the substitution line *CS-1Sl*(*1B*) of wheat.	[108]
AtSerpin1	Mutant *Arabidopsis thaliana* lines overexpressing AtSerpin1 showed protective effect in leaves against photodynamic damage after treatment with acridine orange (AO) photosensitizer and had attenuated cell death by AO and water stress.	[45]
AtSerpin1	Osmotic stress induced the accumulation of *Atserpin1* transcripts and protein complexes of AtSerpin and cognate proteases. In addition, the lines with overexpression of AtSerpin1 showed a protective effect to osmotic stress, while AtSERPIN1 knockout lines exhibited decreased root elongation rate.	[116]
MtSer6	*MtSer6* gene expression increased in response to water stress (drought). In addition, knockdown of *MtSer6* and ferritins genes in response to drought caused increased papain-like cysteine protease activity and free iron levels, early nodule senescence, lower photosynthetic rate and shoot biomass.	[20]
BSZ7 (Serpin-Z7)<br>BSZx (Serpin-Zx)	Serpin-Z7 and Zx were differentially expressed in two contrasting varieties with respect to salinity tolerance. Serpin-Z7 was most accumulated in the salt tolerant variety	[122]
WSZ2a<br>WSZ2b	LC-MS/MS results revealed high abundance, in well-irrigated or stressed plants, of proteins such as Serpin-Z2A and Z2B in an ethyl methanesulfonate (EMS)-induced mutant wheat line (BIG8-1) with greater tolerance to water deficit.	[125]
ApSerpin-ZX	At the transcriptional level Apserpin-ZX was upregulated under abiotic stress caused by saline, oxidative, cold, osmotic stress and cryopreservation in embryogenic callus of *A. praecox*. When serpin was added to the plant vitrification solution, it had a positive effect on the survival rate of cryopreserved callus.	[53]
	Biotic and Abiotic	
OsSRP-LRS	*OsSRP-LRS* expression was induced by inoculation of the necrotrophic fungal pathogen *Rhizoctonia solani*, UV and salt. Furthermore, transgenic RNAi lines with reduced expression of *OsSRP-LRS* showed exaggerated cell death under these conditions.	[19]

AtSRP4; *AtSRP5*	The expression of *AtSRP4* and *AtSRP5* genes was induced after ultraviolet (UV) treatment and inoculation with virulent strains of *Pseudomonas syringae* pv DC3000 carrying the *AvrRpt2* (*Pst-AvrRpt2)* and *AvrB* (*Pst-AvrB*) genes. Furthermore, mutant lines of both serpin genes were more susceptible to cell death by UV and *Pst-AvrRpt2*, and the AtSRP4 overexpression line had lower cell death. *AtSRP4* was induced in leaves by NaCl treatment.	[37]

* The references cited are available in Table S2.
