# Supplementary material for: Plant Serpins: Potential Inhibitors of Serine and Cysteine Proteases with Multiple Functions
Source: Plants (Basel). 2023 Oct 19;12(20):3619. doi: 10.3390/plants12203619 (PMC10609998; doi:10.3390/plants12203619)
Supplement: Supplementary file 1 [file plants-12-03619-s001.zip › Table S5.pdf]

Table S5. Description of the PICO strategy.

Acronym	Definition	Components of the question
P	Population	Plant serpins.
I	Intervention or Interest	Systematize the scientific production of the functionality of serpins in plant systems and the different biotic and abiotic stresses.
C	Comparison	The involvement of plant serpins under biotic and abiotic stresses and their inhibitory potential tested against serine and cysteine proteases.
O	Outcome	Location, functional characterization, inhibitory potential, expression level and biological processes of plant serpins under different conditions.
